# Estimating duration-distance relations in cycle commuting in the general population

**DOI:** 10.1371/journal.pone.0207573

**Published:** 2018-11-16

**Authors:** Peter Schantz, Lina Wahlgren, Jane Salier Eriksson, Johan Nilsson Sommar, Hans Rosdahl

**Affiliations:** 1 Research Unit for Movement, Health and Environment, The Åstrand Laboratory, The Swedish School of Sport and Health Sciences, GIH, Stockholm, Sweden; 2 Unit for Occupational and Environmental Medicine, Department of Public Health and Clinical Medicine, Umeå University, Umeå, Sweden; Faculty of Medicine and Health Sciences, SWEDEN

## Abstract

It is important to estimate the duration-distance relation in cycle commuting in the general population since this enables analyses of the potential for various public health outcomes. Therefore, the aim is to estimate this relation in the Swedish adult population of 2015. For that purpose, the first step was to establishit for adult male and female cycle commuters in Greater Stockholm, Sweden. Whether or not the slopes of these relations needed to be altered in order to make them representative of the general population was evaluated by comparing the levels of maximal oxygen uptake in samples of commuter cyclists and the population. The measure used was the maximal oxygen uptake divided by both the body weight and a cycle weight of 18.5 kg. The body weights in the population samples were adjusted to mirror relevant levels in 2015. Age adjustments for the duration–distance relations were calculated on the basis of the maximal oxygen uptake in the population samples aged 20–65 years. The duration-distance relations of the cycle commuters were downscaled by about 24–28% to mirror levels in the general population. The empirical formula for the distance (*D*, *km*) was based on duration (*T*, *minutes*) · speed (km/min) · a correction factor from cycle commuter to the general population · age adjustment (*A*, *years*). For the males in the general population the formula was: *D* = *T* · 20.76 km/h · 0.719 · (1.676–0.0147 · *A*). For females, the formula was: *D* = *T* · 16.14 km/h · 0.763 · (1.604–0.0129 · *A*). These formulas, combined with distributions of route distances between home and work in the population, enable realistic evaluations of the potential for different public health outcomes through cycle commuting.

## Introduction

Active commuting is a recommended form of physical activity from a public health perspective by, e.g., the World Health Organization [1,2]. It is also regarded as potentially valuable for enhancing air quality and decreasing noise and emissions of green-house gases [3,4]. However, the extent to which active commuting is a feasible strategy within the general population has not been studied. This demands understanding a number of issues, among which temporal and spatial dimensions of active commuting in the population are fundamental. These include: (1) the duration-distance relations in walking and cycling for males and females of different ages, (2) the realistic duration spectrum, and (3) the distribution of distances between home and places of work or study.

**Fig 1:**
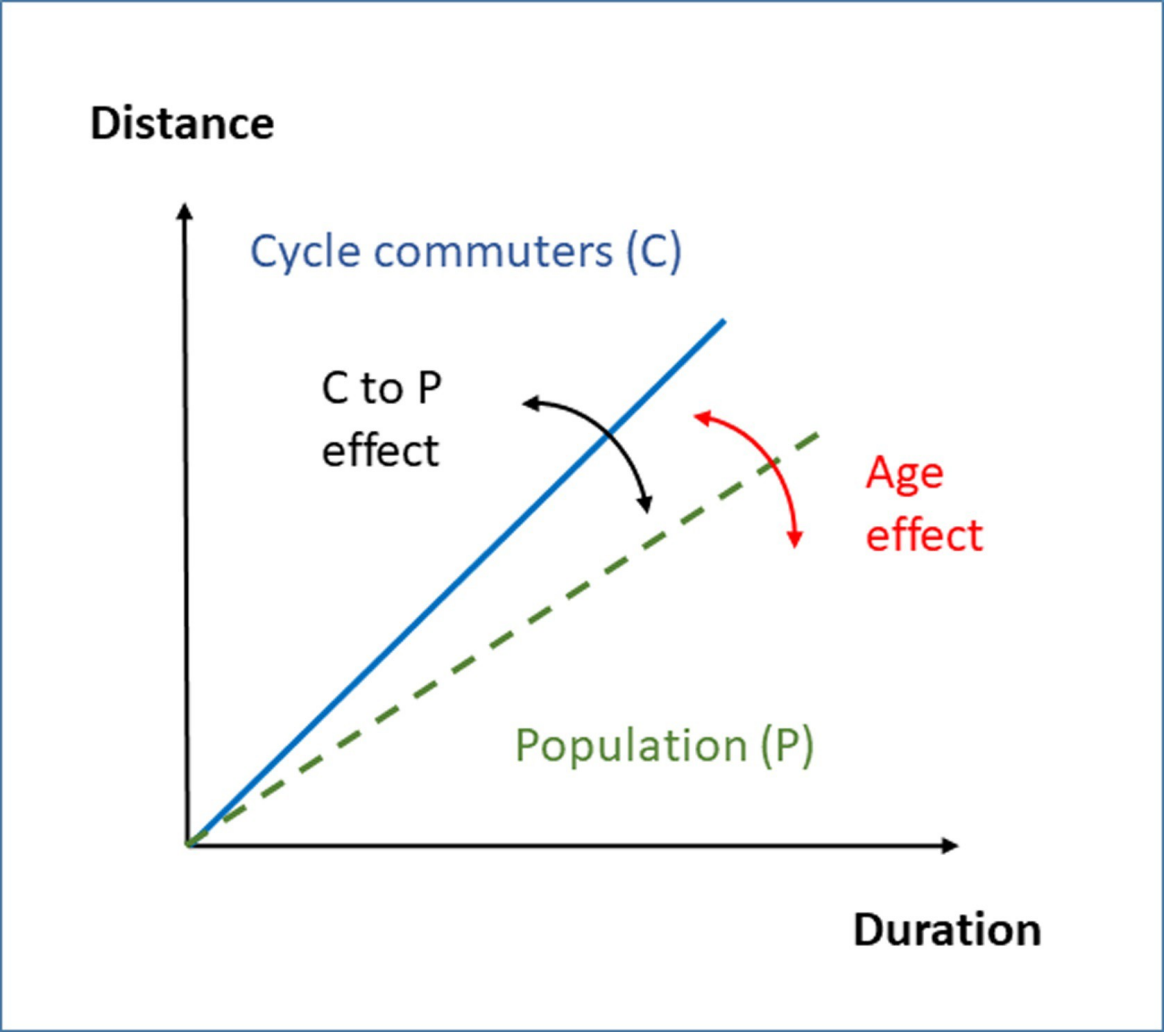
A conceptual model for analyses of the duration-distance relation for cycle commuters and its translation to levels in the general population. The translation of the cycle commuter to general population is indicated as a “C to P effect”, and the modification of the relation depending on age is indicated as an “Age effect”. In the illustration of the model, a lower distance per given duration is indicated for the general population compared to the cycle commuters. However, the placing of that relation in the population relative to the cycle commuters is an open question.

The aim of this study is to estimate the duration-distance relation of cycle commuting in the general population. This demands a number of measures and analyses: (1) valid methods and measurements of durations and distances in current cycle commuters, (2) empirical data from male and female cycle commuting trips, and (3) translation of data from cycle commuters to the male and female general populations, accounting for body weights at the present time (the cycle commuter to general population effect, the C to P effect) and variations in age (the age effect). A conceptual model for these analytical steps is presented in Fig 1.

The aim of this study demands that a number of methodological issues are handled. They will be introduced here. Values for commuting durations and distances should be as correct as possible. This is rather difficult to achieve since self-reported distances among adults of both sexes are generally overestimated, and furthermore the spreading is great [5,6,7]. This is also true for cycle commuters [8]. Even objective methods, such as route choice modeling using geographic information systems (GIS) or global positioning systems (GPS) may overestimate cycling distances by about 5–20% [8]. However, a valid method for cycle commuting is if adult male and female cycle commuters draw their individual routes on maps, and that they are measured with valid distance-measuring instruments. That method is considered to be a criterion method [9].

Obtaining valid trip durations is also a challenge. The preferential rounding off in self-reports to last digit multiples of 5, e.g. [10], indicates problems in itself, and since most studies show that self-reported durations are longer than those derived from GPS, cf. [11], a reasonable interpretation is that, in general, there is a systematic over-reporting of durations associated with the rounding off to last digit multiples of 5. This was also noted when cyclists’ trips were followed on camera. On the other hand, individuals reporting durations with the last digits 1–4 or 6–9 present values that are close to correct [12]. In line with that, estimated cycling velocities are systematically higher (about 1.7 km·h^−1^) for commuter cyclists with last-digit duration reports of 1–4 or 6–9, as compared to multiples of 5 [13]. Duration reports with the last digits of 1–4 or 6–9 are therefore considered to be a close to criterion method.

These methodological advances represent a sound basis for establishing valid duration-distance relations for cycle commuters. The next issues to take into account are the cycle commuter to population effect, and the age effect (cf. Fig 1). The power output in cycling is linearly related to oxygen uptake, e.g. [14], and the maximal oxygen uptake is related to performance in competitive cycling, cf. [15,16]. The relation between maximal oxygen uptake and cycling speed is further supported by sex and age differences in cycle commuting velocities [13], which mirror the differences noted in the maximal oxygen uptake within the population [17], ([18], p. 319). This lends support for handling the cycle commuter to general population effect by comparing the maximal oxygen uptake in cycle commuters and the general population. Furthermore, the age effect within the population can be estimated using the same measure. Applying such a methodological approach has, to our knowledge, not been undertaken before.

With the aim of estimating the duration-distance relation in the general population, the empirical bases were: (1) duration-distance relations and maximal oxygen uptake levels of male and female cycle commuters in the metropolitan area of Greater Stockholm, Sweden, as well as (2) levels of maximal oxygen uptake in the general population, adjusted for the secular weight development in Sweden. The analyses are aimed at establishing formulas for the duration-distance relations with respect to age in the male and female populations in Sweden of 2015.

## Materials and methods

### The duration-distance relation for cycle commuters

#### Participants

The sample is from a larger multidisciplinary research project, Physically Active Commuting in Greater Stockholm (PACS), carried out by the Swedish School of Sport and Health Sciences, GIH, in Stockholm, Sweden. Here only fully active cycling is analysed, i.e., no cycling with electrically assisted cycles (e-bikes) is considered. Ethical approval was obtained from the Ethics Committee North of the Karolinska Institute at the Karolinska Hospital, Stockholm, Sweden (Dnr 03–637), and the participants gave their written informed consent.

Recruitment advertisements for the PACS study were published in the two regional morning newspapers (Svenska Dagbladet and Dagens Nyheter) in May, 2004. No incentives were provided. 2148 individuals responded. They sent in a response coupon from the advertisement with written addresses to their home and place of work or study. The inclusion criteria were: participants should be of a minimum age of 20 years, live in those parts of the County of Stockholm which have “08” as regional digits in their stationary telephone number (which refer to all municipalities in the county, except for the northernmost municipality of Norrtälje), and walk or cycle the whole way to their place of work or study at least once a year. There was no maximum age limit for participation. The information to the commuters emphasized that individuals with very short commuting distances were also welcome to participate. For more details on the selection process, see [19]. The participants recruited with this sampling method compares well with age, economical and educational background of the cycle commuters in the same region, but they appear to cycle for longer durations ([20], p. 109–110). This study is based on the subsample of cycle commuters that used the last digits 1–4 or 6–9 in their trip duration reports (n = 455). The rationale for this is that they represent near-valid duration reports [12,13].

#### Questionnaire and administration

A paper-based questionnaire created for the PACS Study (PACS Q1) was sent out in September, 2004. PACS Q1 is self-administered, written in Swedish, contains 35 items, and it takes about 15 minutes to respond to. The PACS Q1 questionnaire in the original version in Swedish, and in a version translated into English, are provided in S1 and S2 Methods, respectively. The information used in this study was gender, height, weight, year of birth, cycle commuting durations to work, the number of gears of the bicycle and level of physical activity during working hours.

#### Maps and route distance measurements

An individually adjusted map was prepared for each participant. It was based on the respondents’ written home and place of work or study addresses. An instruction on how to draw their own cycle commuting routes was included (see S3 Methods for the original Swedish version, and S4 Methods for the version translated into English). Before filling out the route on the map, the participants were asked to cycle their route once, noting their choice of route and the street names. They were also asked to pay particular attention when marking their route in case it followed places outside the printed street network, such as parkways. Their homes were marked with the capital letter B (“B” for bostad, i.e. home in Swedish) and their place of work or study with a small box. Both the routes to and from the place of work or study were marked on the maps. In this study, only the routes from home to the place of work or study were used. The route distances were measured twice with a digital curvimetric instrument (Run Mate Club, CST/Berger, Watseka, IL, USA), and expressed in full meters, using a criterion method with high validity and reproducibility. For further methodological details, see Schantz and Stigell [9] and Stigell and Schantz [19].

#### Commuting durations

Participants were asked to record and state the durations (in hours and full minutes) of their commuting trips from home to the place of work or study on a normal day when no other errands were undertaken. The commuting could take place at any time of the year, but given the months that the PACS Q1 was sent out, the values presented are based on commuter cycling in predominantly September and October, and the commuting occurred during daylight conditions, cf. [19].

#### Cycling environment

The study area of PACS is the metropolitan region of Greater Stockholm, with about 1.9 million inhabitants. It consists of inner urban, suburban, and rural areas. Its geographic boundaries and characteristics have been described elsewhere [21, 22]. It is important in this context that these areas represent distinctly different settings with regard to many route environmental variables [22] and that the cycling velocities have been shown to be about 0.7 km · h^−1^ higher in the suburban–rural *versus* the inner urban areas when controlling for a number of variables [13]. Each cyclist´s origin and destination for the commute were therefore located in relation to the inner urban *versus* the suburban–rural area, hereafter referred to as the suburban area. This was based on the postal area codes in the addresses to the participants’ homes and places of work or study. The cycle commuters were divided into three groups: (0) both origin and destination in the inner urban area, (1) one of them in the inner urban and the other in the suburban area, and (2) both of them in the suburban area.

Another factor that can affect the duration-distance relation is the degree of hilliness. Generally, the landscape in Greater Stockholm is rather flat, but, rather frequently, it includes smaller and gentle slopes, and occasionally also more demanding hills of up to about 15 m elevation. However, this was not a factor that we specify in more detail or make use of in the analyses.

#### The relation between age and the duration–distance relationship

Whether there was a systematic relation between age and the cycled trip duration, which could affect the distances, was checked for males and females, respectively (see Statistics).

#### Characteristics of the participants

The characteristics of the participants who contributed to establishing the duration-distance relation are given in Table 1. The number of gears of the cycles were categorized into 0, 2–4, and 5 and more gears. The percentage of these were for the males; 6, 16 and 78%, and for the females; 11, 33 and 56%.

**Table 1:** Participant characteristics in the sample of cycle commuters on which the duration-distance relations are based (mean value, standard deviation and 95% confidence interval).

Cycle	Age	Height	Weight	BMI	Duration	Distance	Velocity	Cycling
commuters	yrs	cm	kg	kg·m^−2^	min	km	km·h^−1^	environment*
**Males**	45.8	180	78.1	24.0	23.9	7.91	18.4	1.18
(n = 175)	11.6	6.8	8.9	2.4	16.2	6.5	4.3	0.77
	44.0–47.5	179–181	76.8–79.4	23.6–24.4	21.5–26.4	6.97–8.86	17.8–19.1	1.07–1.30
**Females**	46.9	168	64.5	22.8	16.6	4.22	14.4	1.20
(n = 280)	10.7	6.3	9.2	2.9	10.3	3.3	3.9	0.87
	45.6–48.1	167–169	63.4–65.6	22.5–23.2	15.4–17.9	3.83–4.61	13.9–14.8	1.10–1.30

Note: * Cycling environment: 0 = inner urban; 1 = inner urban-suburban; 2 = suburban.

### Estimation of the maximal oxygen uptake

#### Recruitment and selection of cycle commuters

The initial recruitment steps for the PACS Study are described in the previous section. The process of selecting participants for the exercise physiologal tests was divided into several steps. The first was to divide the respondents into categories based on their mode of either cycling or walking, or combining both modes. For details on the selection and categorization processes, see [19]. The single-mode cyclists constitute the category of respondents who only cycled to work. From that category, representative subsamples of male and female single-mode cyclists were selected for this study. They had ages and route distances close to the median values of the male and female single-mode cyclists, and they rated their daily professional jobs as physically light or very light.

A letter describing the physiological studies and test procedures was sent in 2006 to the cyclists who fulfilled the criteria. The recipients were first asked if their previously described cycling route was still valid, or if a comparable distance time-wise (duration = ± 5–10 min duration) was applicable at the present time. They were then asked to respond to a declaration of health concerning (1) medication and for which kind of illness, (2) whether they had any palpitations, chest pain, or abnormally heavy breathing during exercise, (3) if they had high blood pressure, and (4) if they had recently avoided or discontinued exercise for reasons of injury or health. The letter emphasized the right to terminate the tests at any time and without having to give a reason. A signed informed consent to participate was returned.

Based on this information, individuals with invalid route distances, as well as those with high blood pressure or were on medication that could affect the normal heart rate were excluded. Anyone on medication with risks for strong side effects was also excluded. We checked the effect of each drug in the official pharmacy guide for medical personnel in Sweden (Farmaceutiska Specialiteter i Sverige, FASS), and consulted a medical doctor specialized in circulation physiology if needed. We contacted the remaining cyclists by telephone to answer any potential questions and to book their test times. Telephone contacts continued until we had 10 women and 10 men who fulfilled the criteria and were willing to participate. For characteristics of the participants, see Table 2. It is based on the same type of measurements as described in the previous sections, except for the cycle trip durations, which were measured in full minutes by two of the authors (PS and JSE), being present at the cyclists´ origin and destination points for the cycling trip. The differences noted in velocity and distance between the sexes in both Tables 1 and 2 are in line with what could be expected based on a combined effect of duration and sex, cf. [13].

**Table 2:** Characteristics of the commuter cyclists participating in the estimations of maximal oxygen uptake (mean value, standard deviation and 95% confidence interval).

Cycle	Age	Height	Weight	BMI	Duration	Distance	Velocity	Cycling
commuters	yrs	cm	kg	kg·m^−2^	min	km	km·h^−1^	environment*
**Males**	42.8	185	84.8	24.6	29.1	9.61	20.1	1.10
(n = 10)	4.3	7.2	12.7	3.1	7.2	2.2	2.7	0.32
	39.7–45.9	180–191	75.7–93.9	22.4–26.8	24.0–34.2	8.05–11.2	18.2–22.0	0.87–1.33
**Females**	43.3	170	65.8	22.7	22.2	6.51	17.5	1.10
(n = 10)	2.5	5.3	7.6	2.5	3.5	1.5	2.8	0.74
	41.5–45.1	167–174	60.4–71.3	20.9–24.5	19.7–24.7	5.43–7.60	15.5–19.6	0.57–1.63

Note: * Cycling environment: 0 = inner urban; 1 = inner urban-suburban; 2 = suburban.

#### Recruitment and selection of samples from the general Swedish population in 1990 and 2000

The estimated levels of maximal oxygen uptake in the general Swedish population are based on the original data from the LIV Studies, which are two separate national cross-sectional surveys, one conducted in 1990–1991 (LIV 1990)[23] and one in 2000–2001 (LIV 2000)[24]. In both surveys, inhabitants aged 20–65 years were drawn from the Swedish Population and Address Registry (SPAR). In 1990–1991, a total of 2400 inhabitants, and in 2000–2001, a total of 2000 inhabitants were drawn. In the 1990–1991 group, 1222 inhabitants and, in the 2000–2001 group, 500 inhabitants were tested for maximal oxygen uptake and had weight and age data. The data collection was done at test centres manned by trained personnel from either a national fitness organisation (LIV, 1990 and 2000; Svenska Korporationsidrottsförbundets länsförbund) or a commercial health company (LIV 2000; AB Previa). Other measures of maximal oxygen uptake, anthropometric, questionnaire, and test data from these studies have been published elsewhere [23,24,25]. The LIV data was fully anonymized before we received them.

#### Instrumentation

The types of instrumentation used for the Åstrand cycle ergometer test were typical standard pieces of equipment, whereas the specific brands described here apply to the tests of the cycle commuters by the trained staff at the Swedish School of Sport and Health Sciences, GIH.

#### Cycle ergometer

A manually braked pendulum cycle ergometer (828E Monark Exercise AB, Varberg, Sweden) was used. A digital metronome (DM70 Seiko S-Yard Co. Ltd, Tokyo, Japan) helped the subjects to maintain the correct cadence while cycling.

#### Heart rate

During execution of the exercise protocol, heart rate was measured and averaged every 15 s, using a Polar Electro S610i heart rate monitor (Polar Electro Oy, Kempele, Finland) with a Polar Wearlink 31 transmitter (Polar Electro Oy, Kempele, Finland).

#### Exercise test methods

All participants were weighed standing on a calibrated standard scale, to the nearest 0.1 kg, and wearing lightweight clothes. Body height was measured with a calibrated stadiometer to the nearest 0.1 cm.

#### The cycle commuter studies

This exercise protocol was done to mimic, as closely as possible, the procedures in the LIV Studies [23,24,25]. The participants were asked to follow these procedures before the test occasion: (1) not to engage in any vigorous exercise for 24 hours beforehand, (2) not to cycle to the test place, (3) to refrain from smoking, and taking snuff (i.e. smokeless tobacco) for at least one hour before arrival at the laboratory, (4) not to eat a light meal at least one hour before the tests, (5) not to eat a large meal at least three hours before the tests, (6) to avoid stress, and (7) to cancel the test if they had fever, an infection, or a cold. This protocol was slightly more developed than the one used in the LIV Studies, which only handled Nos. 1, 3, and 4 above, and in approximately the same manner [25].

Before each experiment, the scale was zeroed while each subject sat upright on the saddle with his or her feet resting on the frame between the pedals, and hands resting on the handle bars. The saddle height was adjusted so that the participant’s knees were slightly flexed when the feet were on the pedals in their lowest position. The handle bars were adjusted to allow the participants to sit in an upright position. The work rate was controlled every minute by checking the cadence of the participant and the braking force as indicated on the pendulum scale.

The participants cycled in an upright position at three different work rates: males, 100, 150, and 200 watt (W) and females, 50, 100, and 150 W, at a cadence of 50 revolutions per minute. At each work rate, the participant cycled until steady-state (approx. 6 minutes), after which the resistance was increased. The third work rate was increased to only 125 W or 175 W for women and men, respectively, if, after the second work rate, the subject’s heart rate was higher than 150 beats per minute (bpm) and their perceived rate of exertion exceeded 15 [26]. The first test load with accepted steady-state heart rate values (range, 120–170 bpm) was used. Maximal oxygen uptake (L · min^−1^) was calculated based on submaximal ergometer work rates, the steady-state heart rate and an age correction factor according to the Åstrand-Ryhming nomogram ([14], as modified in [17]; see [27], pp 281, 287).

#### The population studies (LIV 1990 and LIV 2000)

Two submaximal work rates (for most individuals, in the range of 75–175 watt), with a cadence of 50 revolutions per minute, were used. Unlike in previous reports from the LIV Studies [23,24,25], the first test load with accepted steady-state heart rate values in the range 120–170 beats · min^−1^ was used for the calculations presented here. Maximal oxygen uptake (L · min^−1^) was calculated based on submaximal ergometer work rates and the steady-state heart rate, as well as a correction factor for age, according to the Åstrand-Ryhming nomogram ([14], as modified in [17]; see [27], pp 281, 287).

### Estimations of the body weight development in the general Swedish population during 1988–2013

Data on the secular development in body weight were gathered from multiple samples derived from three different sources: (1) The LIV 1990 and LIV 2000 studies at the Swedish School for Sport and Health Sciences, GIH [23,24] samples from 1990/91 were analyzed as “1990” and samples from 2000–2001 were analyzed as “2000”), (2) the nationwide investigations, Swedish Survey of Living Conditions (ULF)(samples from 1988–89 were analyzed as “1988”, 2008–09 as “2008”, and 2010–11 as “2010”), sampled by Statistics Sweden [28], as well as (3) the National Public Health Enquiry (FHE)(samples from every year from 2004 to 2013), sampled by the Public Health Agency of Sweden)[29].

The LIV Studies contain measured weight, whereas the ULF and FHE Studies rely on self-reported weight. Since these sources presented their data in slightly different fashions, new categories were created in order to be able to combine the data. Five age categories were formed (Table 3) and the categories were treated as being representative of the age groups 20–29, 30–39, 40–49, 50–59, and 60–69. For this reason, the original 5-year categories in the LIV and ULF Studies were weighed depending on the sample sizes so as to create the new categories with 10-year spans. Thereby, the LIV and ULF data could be transformed into fully comparable age spans, whereas the FHE data were fitted to the age categories as shown in Table 3. The LIV, ULF and FHE data were fully anonymized before we received them.

**Table 3:** Age categories used for the analytical purposes and the matching of data from different population studies.

Age category yrs	Population studies		
	LIV	ULF	FHE
20–29	20–29	20–29	16–29
30–39	30–39	30–39	30–44*
40–49	40–49	40–49	30–44*
50–59	50–59	50–59	45–64
60–69	60–65	60–69	65–84

Note: * The same data were used twice. For abbreviations, see the text.

The accumulated data for males and females from the different investigations and years were analyzed for secular trends with sample size weighed regression analyses of the mean values for different age groups during the period 1988–2013 (Table 4). The rationale for using a linear regression in our study is based on the the linear increases in weight, overweight and obesity in Sweden, many Western countries and globally during this time period [30,31].

**Table 4:** Relation between body weight and calendar year in different age categories for males (n = 72516) and females (n = 77722)(y-intercept and regression coefficient with 95% confidence interval, CI).

Population samples Sex and age category	Body weight kg			Calendar year Anno Domini			R^2^
**Males**	**y-intercept**	**95% CI**	**P-value**	**regression** **coeffient**	**95% CI**	**P-value**	
20–29 (n = 9330)	-134	-333–66	0.173	0.11	-0.01–0.21	0.040	0.29
30–39 (n = 12,072)	-470	-624 –-317	0.000	0.28	0.20–0.35	0.000	0.82
40–49 (n = 18,477)	-365	-549 –-181	0.001	0.22	0.13–0.32	0.000	0.68
50–59 (n = 18005)	-457	-572 –-342	0.000	0.27	0.21–0.33	0.000	0.89
60–69 (n = 14,632)	-322	-565 –-78	0.013	0.20	0.08–0.32	0.003	0.50
**Females**							
20–29 (n = 11,356)	-204	-299 –-110	0.000	0.13	0.09–0.18	0.000	0.74
30–39 (n = 14,732)	-418	-581 –-255	0.000	0.24	0.16–0.32	0.000	0.76
40–49 (n = 14,911)	-268	-362 –-174	0.000	0.17	0.12–0.21	0.000	0.81
50–59 (n = 20,981)	-346	-440 –-251	0.000	0.21	0.16–0.25	0.000	0.87
60–69 (n = 15,742)	-177	-299 –-54	0.008	0.12	0.06–0.18	0.001	0.59

### Estimating the duration-distance relation for the general Swedish population in 2015

#### Addition of body weight and calculation of the relative maximal oxygen uptake

The secular trends in body weight were used to adjust the body weights in the general population- based LIV 1990 and 2000 Studies up to the year 2015 (Tables 5 and 6). The average gain in weight in each age span was added to the individual weights of each participant. Thereafter, the individual maximal oxygen uptake values from the LIV Studies (L · min^−1^) were transformed into relative maximal oxygen uptake levels on the basis of both the estimated body weight (bw) in the year 2015 and a cycle weight (cw) of 18.5 kg (mL · (bw + cw kg)^−1^ · min^−1^). The reason for including cycle weight is its possible effects on energy demands in cycling due to the rolling resistance and the gravitational effect on ascents, cf. [32]. The cycle weight used (18.5 kg) is the median weight for male and female cycles sold in the Stockholm region during several years [13].

**Table 5:** Original anthropometric data for males and females in the LIV 1990 Study and the estimated added weight during 1990–2015, as well as new BMI values in 2015 (mean values).

	Males					Females				
	1990			2015		1990			2015	
Age yrs	Height cm	Weight kg	BMI kg·m^−2^	Weight gain kg	BMI kg·m^−2^	Height cm	Weight kg	BMI kg·m^−2^	Weight gain kg	BMI kg·m^−2^
20–29	180	76	22.6	2.7	24.3	167	64	22.9	5.7	24.9
30–39	180	80	24.5	7.0	26.7	167	65	23.5	6.0	25.7
40–49	178	81	25.5	5.5	27.3	165	66	24.1	4.2	25.7
50–59	177	80	25.7	6.8	27.8	163	68	25.6	5.2	27.6
60–65	176	82	26.4	5.0	28.1	162	69	26.5	3.0	27.6

Note: BMI data in 1990 are calculated from average height and weight data in Engström et al., ([22], p. 66).

**Table 6:** Original anthropometric data for males and females in the LIV 2000 Study and the estimated added weight during 2000–2015, as well as new BMI values in 2015 (mean values).

	Males					Females				
	2000			2015		2000			2015	
Age yrs	Height cm	Weight kg	BMI kg·m^−2^	Weight gain kg	BMI kg·m^−2^	Height cm	Weight kg	BMI kg·m^−2^	Weight gain kg	BMI kg·m^−2^
20–29	180	81	25.0	1.6	25.5	167	64	23.2	1.9	23.9
30–39	182	85	25.7	4.2	27.0	167	67	23.8	3.6	25.1
40–49	181	86	26.1	3.3	27.1	166	68	24.6	2.5	25.5
50–59	179	86	26.9	4.0	28.2	165	68	25.0	3.1	26.2
60–65	176	84	26.8	3.0	27.9	165	72	26.8	1.8	27.4

Note: All data from 2000 are based on Ekblom-Bak et al., ([24], p. 23–24).

#### Estimation of the cycle commuter to population effect

Duration-distance relations regarding males and females in the general population were estimated on the basis of duration-distance relations in cycle commuters and a comparison of maximal oxygen uptake between these different groups of individuals. Based on the estimated linear association between maximal oxygen uptake and age in the population, the maximal oxygen uptake was predicted for the male and female cycle commuters who also were measured for their maximal oxygen uptake. The predicted maximal oxygen uptake then corresponded to the average maximal oxygen uptake within the general population, at the corresponding individual ages. Thereafter, the cycle commuter-to-population effect was estimated as the average ratio between the predicted maximal and the measured maximal oxygen uptake of the 10 male and 10 female cycle commuters, respectively. The estimated duration-distance relations among the larger sample of male and female cycle commuters, respectively, were then downscaled by these ratios.

#### Estimation of the age effect

The effect of age on the duration-distance relation was estimated on the basis of the age associated decrease in maximal oxygen uptake in samples from the general population. Predictions of maximal oxygen uptakes at the evenly distributed ages 25, 35, 45, 55, and 65 were compared with predictions of maximal oxygen uptake for the mean ages in the large sample of cycle commuters on which the duration-distance relation was based (45.8 years for males and 46.9 years for females). The relative effect of age on the maximal oxygen uptake was then calculated as the ratio between maximal oxygen uptakes at the specific and mean ages of the cycle commuting males and females, respectively.

The age effect on the duration-distance relation was then estimated by an estimated linear relation between these ratios and age. The duration-distance slope was multiplied by this linear regression equation including an intercept (allowing the ratios to have a non-zero intercept with age) and a slope (estimating the decreasing ratio between oxygen uptake in the general population with increasing age). The rationale for treating the age effect between 20 to 70 years of age in maximal oxygen uptake as a linear function comes from several studies (cf. [18], p. 306), including this study.

### Statistical analyses

Data were entered in the Statistical Package for the Social Sciences and analyzed in version 24.0 (IBM SPSS Inc., Somers, NY, USA). The values are stated as means, standard deviations (SD), and 95% confidence intervals (CI) unless stated otherwise. Linear regression was used to estimate the relation between duration and distance among males and females, respectively, assuming an intercept of 0. Whether there was a relation between cycling duration and age was checked with linear regression. The secular trend in body weight were estimated using linear regression where each data point was weighted according to the number of individuals it represented. The cycle commuter to population effect (cf. Fig 1) was calculated through comparing each single cycle commuter’s estimated maximal oxygen uptake in relative terms with the value at the corresponding age in the general population using fitted linear regression model equations for the male and female populations, respectively. The respective ratios for the male and female cyclists were analyzed with the one-sample T-test, where the null hypothesis was that ratios are equal to 1. The level of statistical significance was set to p<0.05.

## Results

### The duration-distance relation for cycle commuters

**Fig 2:**
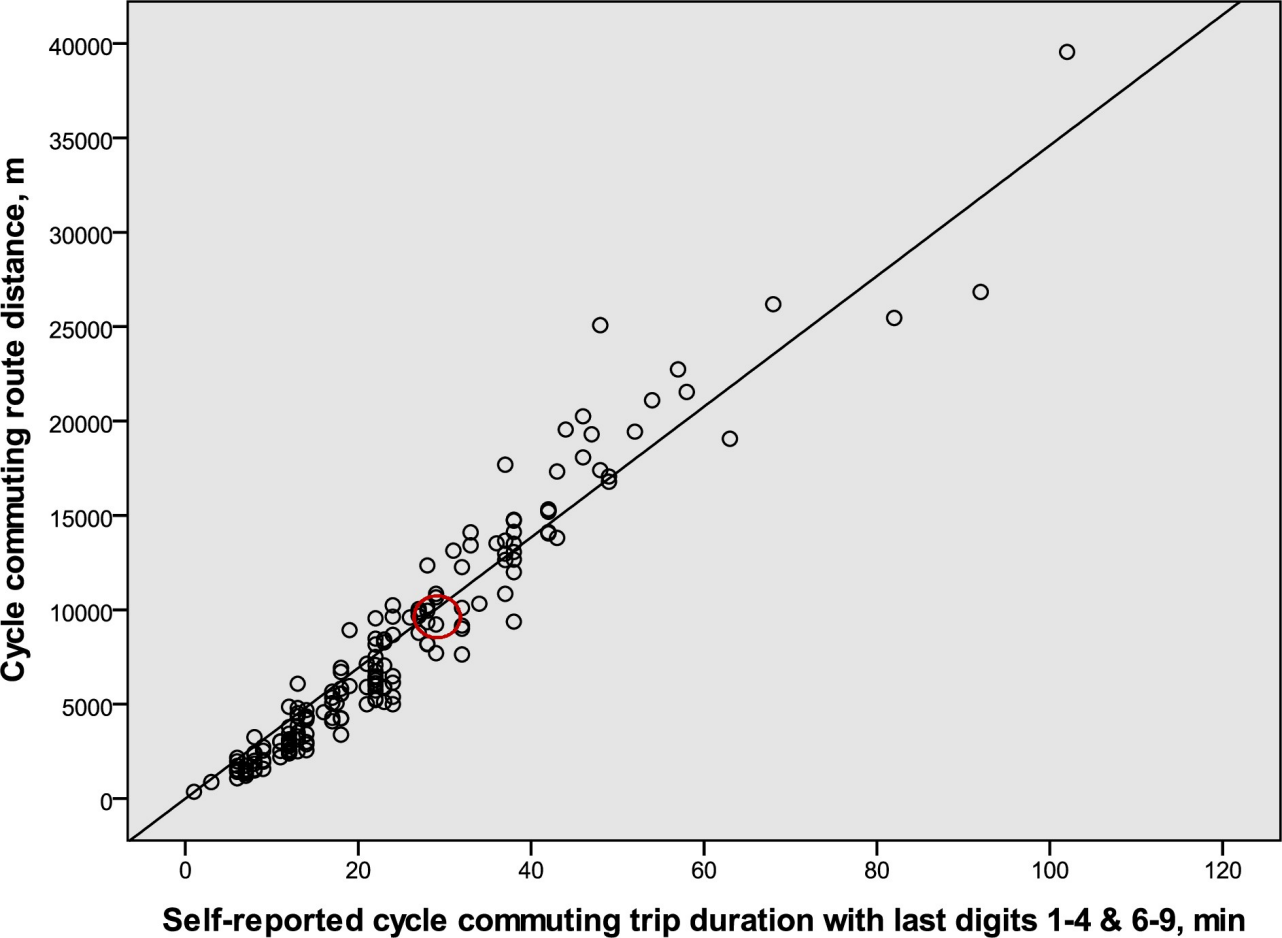
The relation for male cycle commuters between durations and route distances for cycle commuting (n = 175). Only self-reported durations with the last digits 1–4 or 6–9 are represented in the figure. The center of the red circle represents the corresponding mean values for the sample of cycle commuters that were used to calculate the cycle commuter to population effect (see Fig 1).

**Fig 3:**
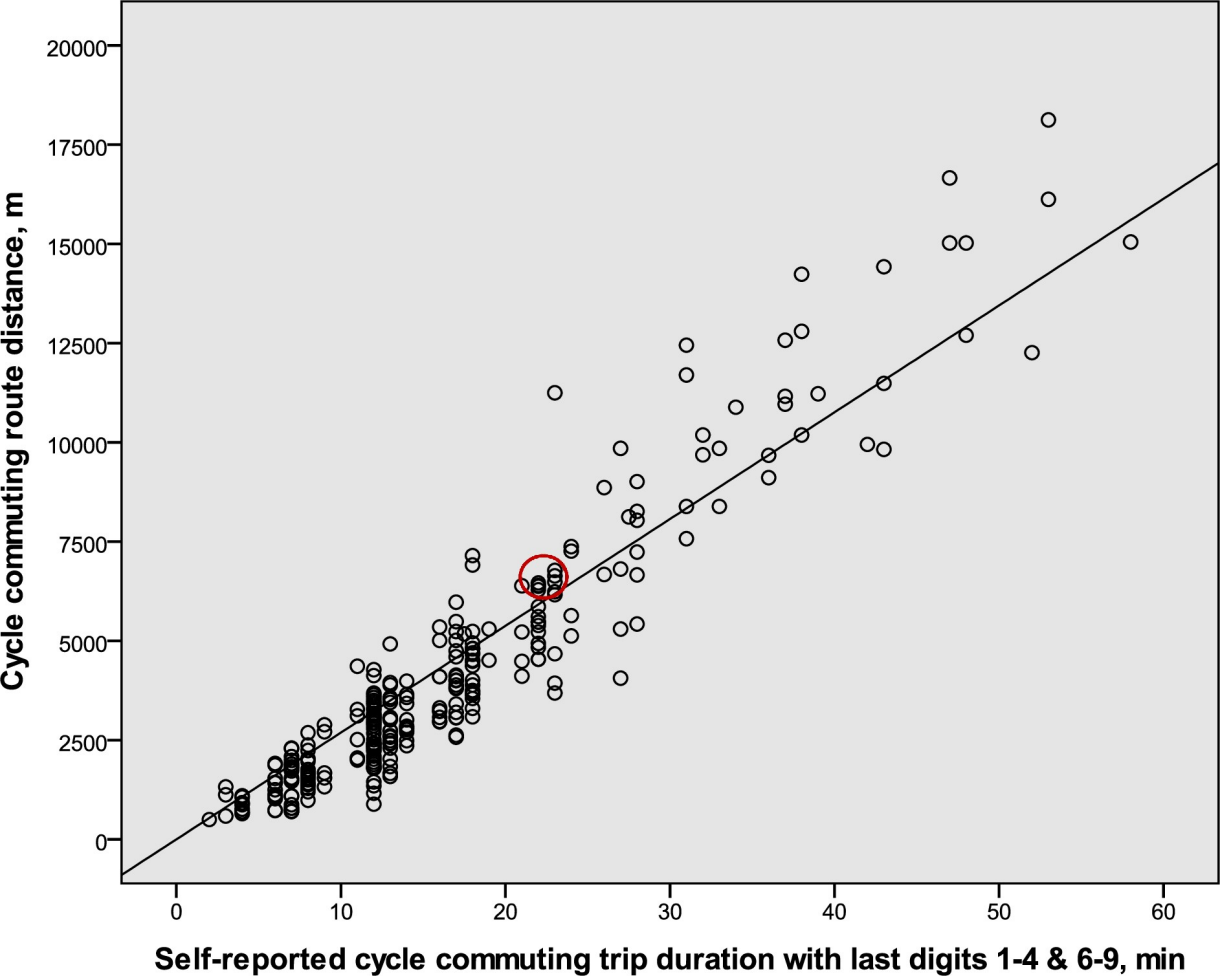
The relation for female cycle commuters between durations and route distances for cycle commuting (n = 280). Only self-reported durations with the last digits 1–4 or 6–9 are represented in the figure. The center of the red circle represents the corresponding mean values for the sample of cycle commuters that were used to calculate the cycle commuter to population effect (see Fig 1).

The regression equations for the duration-distance relation, assuming a zero intercept, were, for the males, distance (m) = 346 (95% CI: 337–355) · duration (min) (R^2^ = 0.97, p < 0.001, n = 175), which corresponds to the velocities 20.76 km·h^−1^ (95% CI: 20.2–21.3). For the females, distance (m) = 269 (95% CI: 262–276) · duration (min) (R^2^ = 0.95, p < 0.001, n = 280), which corresponds to the velocities 16.14 km·h^−1^ (95% CI: 15.7–16.6). Regression analyses (not shown) revealed that there was no significant relation between cycling duration and age in males or females, respectively. The individual values for the males and females are illustrated in Figs 2 and 3. To indicate the corresponding mean values for the sample of cycle commuters that were used to calculate the cycle commuter to population effect (the C to P effect) illustrated in Fig 1, circles in red are placed in Figs 2 and 3. The mean values are in the center of those circles (cf. Table 2).

### Estimated levels of maximal oxygen uptake in the Swedish population in 2015 and in the cycle commuters

**Fig 4:**
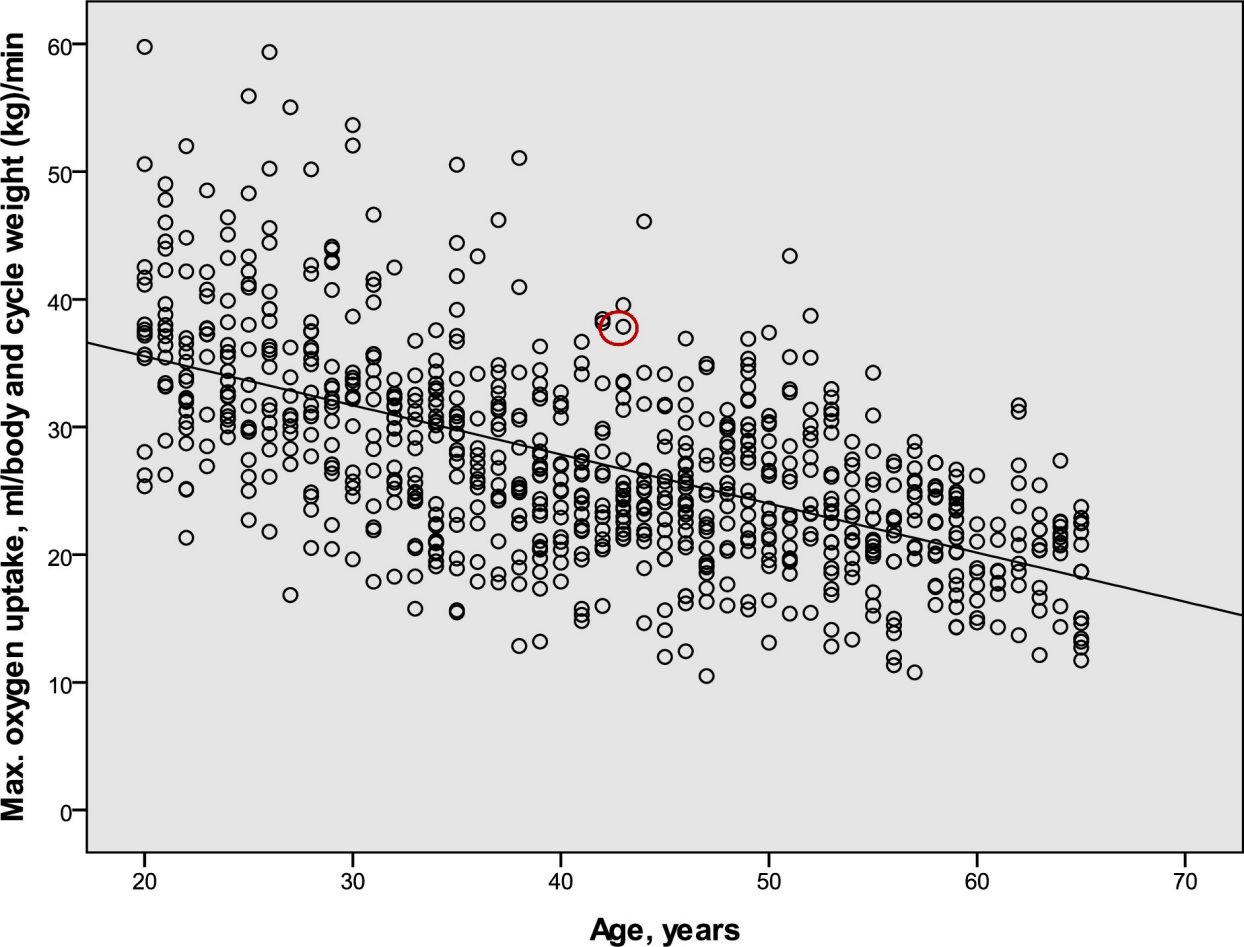
The relation in a male population sample between age and estimated maximal oxygen uptake (n = 803). The maximal oxygen uptake is expressed as mL O_2_ · (body weight + cycle weight, kg)^−1^ · min^−1^. The center of the red circle represents the corresponding mean value for the sample of cycle commuters that were used to calculate the cycle commuter to population effect (see Fig 1).

**Fig 5:**
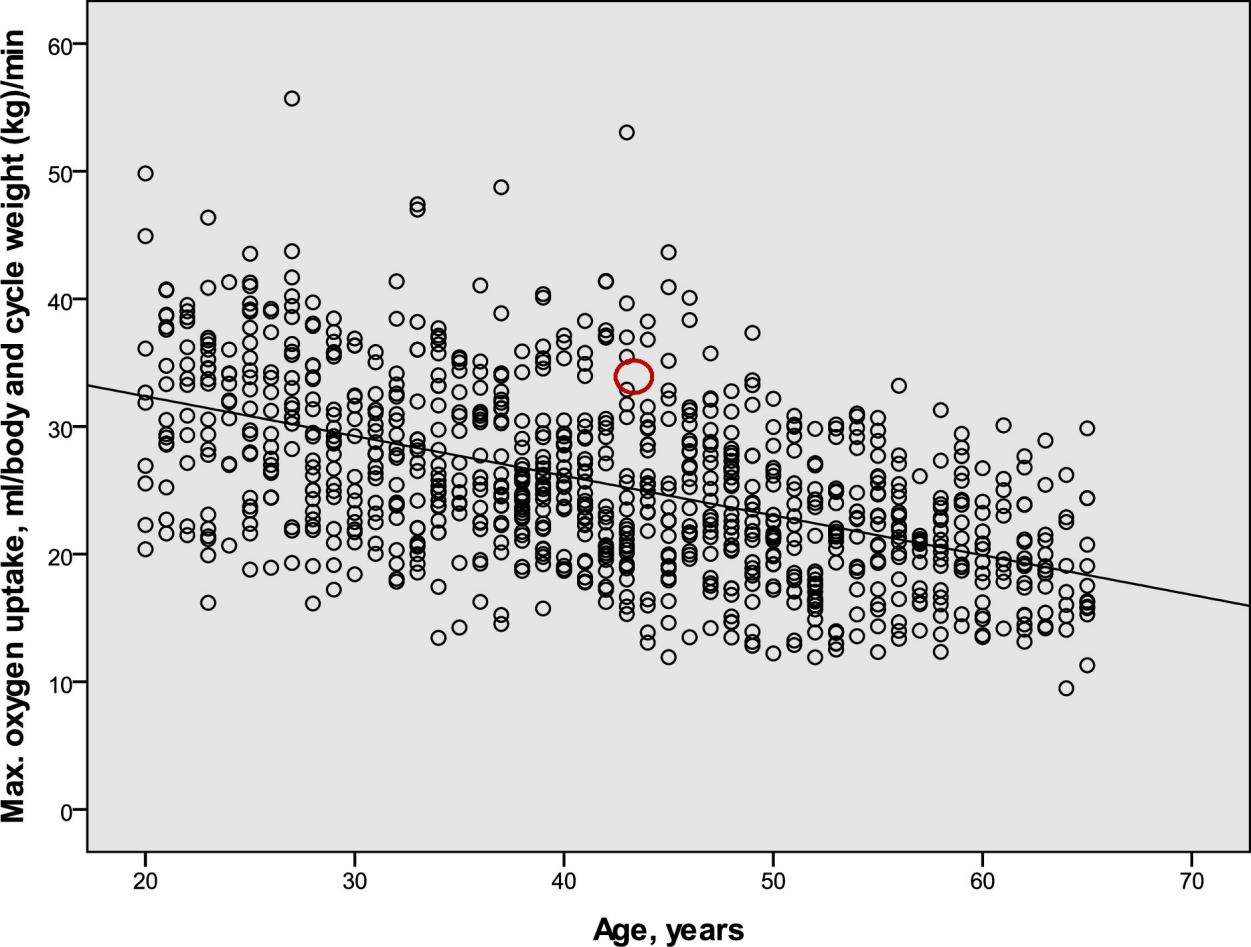
The relation in a female population sample between age and estimated maximal oxygen uptake (n = 919). The maximal oxygen uptake is expressed as mL O_2_ · (body weight + cycle weight, kg)^−1^ · min^−1^. The center of the red circle represents the corresponding mean value for the sample of cycle commuters that were used to calculate the cycle commuter to population effect (see Fig 1).

The relations between age and estimated maximal oxygen uptake relative to body and cycle weight (mL · (bw + bcw kg)^−1^ · min^−1^) in Swedish males and females in 2015 are given in Table 7. They are based on measurements in 1990 and 2000, but with the secular body weight increase (cf. Tables 4, 5 and 6) added so as to mimic the 2015 population levels. The equations based on the updated 1990 and 2000 data, respectively, were similar (not shown). The individual values for the combined updated 1990 and 2000 data for males and females are illustrated in Figs 4 and 5.

**Table 7:** Regression equations for the relation between age and the maximal oxygen uptake relative to body and cycle weight (mL · (bw + cw kg)^−1^ · min^−1^) in Swedish males and females based on measurements in LIV 1990 and 2000, but with body weights extrapolated to population levels in 2015.

Population samples	Maximal oxygen uptakemL · (bw + cw kg)^−1^ · min^−1^			Age years			R^2^
	y-intercept	95% CI	P-value	Regression coefficent	95% CI	P-value	
**Males** (n = 803)	43.2	41.7–44.8	p < 0.001	-0.38	-0.42 –-0.35	p < 0.001	0.36
**Females** (n = 919)	38.6	37.1–40.0	p < 0.001	-0.31	-0.34 –-0.28	p < 0.001	0.27

The estimated levels of maximal oxygen uptake in the sample of cycle commuters was 37.9 ± 5.2 mL · (bw + cw kg)^−1^ · min^−1^ (mean ± SD)(95% CI: 34.2–41.6) for the males (n = 10), and 33.5 ± 4.5 (95% CI 30.3–36.7) for the females (n = 10). To relate the levels noted in the cycle commuters to those found in the population, the average levels are indicated in Figs 4 and 5. Based on each individual cycle commuters´ age, his or her estimated level of maximal oxygen uptake was compared to the regression lines for the population samples (Table 7). The males had 40.9 ± 16.5% higher (mean ± SD)(95% CI: 29.1–52.7)(p<0.001) levels than those expressed by the regression line for the population. The corresponding levels for the females were 33.5 ± 20.7% higher (mean ± SD)(95% CI: 18.7–48.3)(p<0.001).

### The estimated duration-distance relation for the general Swedish population in 2015

Based on the relative differences in estimated maximal oxygen uptake between the cycle commuters’ individual values and the age-matched values based on the regression equations for the general population samples (Table 7), a correction factor for the slope of the duration-distance relation for the cycle commuters (the cycle commuter to population effect (C to P effect), cf. Fig 1), was estimated to be 0.719 ± 0.087 (95% CI: 0.657–0.781) among males and 0.763 ± 0.104 (95% CI: 0.689–0.838) among females. Both these ratios were significantly different from 1.0 for both sexes (p <0.001).

The age correction factors were calculated on the basis of the regression equations between maximal oxygen uptake for males and females in general population samples (Table 7). For males, the age correction factor was (1.676–0.0147 · *A*), where *A* stands for age (years) and, for the females, it was (1.604–0.0129 ·*A*).

Incorporating the age effect and the cycle commuter to the population effect resulted in empirical formulas for the duration-distance relation in the 2015 Swedish population. The formula estimating the distance (*D*, *km*) based on duration (*T*, *minutes*) and age (*A*, *years*) for males in the population in the age span of 20–65 years was: *D* = *T* · 20.76 km/h · 0.719 · (1.676–0.0147 · *A*), where the factor 0.719 reflects the cycle commuter-to-population effect. For females in the same age span, the formula was: *D* = *T* · 16.14 km/h · 0.763 · (1.604–0.0129 · *A*), where 0.763 reflects the cycle commuter to general population effect.

## Discussion

The main result of this study consists in the formulas for the duration-distance relation (DDR) of cycle commuting with sex and age (20–65 years) in the general Swedish population of 2015. They enable more realistic evaluations of distances that can be cycled within certain durations in the general population, and induce various health and environmental outcomes, for example when switching from car to cycle trips, cf. [33]. Thus, the formulas can serve multiple forms of public health-related evaluations. Given that they represent novelties, we cannot discuss them in relation to other corresponding results. However, the bases for the different stages in the formation process and issues of external validity will be discussed below.

The first step was to establish the DDR for existing cycle commuters. It is important for its validity that route distances were measured with a criterion method based on adult cycle commuters of both sexes [9] and that the last digits in the duration reports were 1–4 or 6–9, which stand for values that are close to being valid [12, 13] and thereby constitute a close to criterion method. Furthermore, it was important to note that the age was stable with different durations in both males and females, and was thereby not a factor affecting the slopes of the DDR.

Comparing the male and female regression equations for DDR, a distinct difference emerges. This is most likely due to objective factors connected to biological sex, cf. [13,32,34] on the one hand, and subjective on the other as well as cultural gender dimensions.

The DDR slopes represent mean values for the whole range of durations. However, if it is of importance for any analyses, note that shorter durations than about 30 minutes for females and 40 minutes for males are, on average, associated with slightly shorter distances and that durations above that time are associated with slightly longer distances than the formulas indicate (cf. the regression equations and the individual values in Figs 2 and 3).

In the samples of cycle commuters in which maximal oxygen uptake was estimated the mean values for the DDR were close to the regression line for the greater sample (cf. Figs 2 and 3). Thus, they were representative for the overall DDR of the cycle commuters.

The next part of the formula relates to the possible need to adjust the DDR slope of the cycle commuters to general population levels. The measure for determining whether this need existed was to compare the maximal oxygen uptake per minute divided by body and cycle weight (18.5 kg) between the samples. The importance of adding cycle weight to the body weight is that cycling involves moving both a person and a vehicle and that the total weight can affect the energy demands coupled with the rolling resistance and gravitational ascents [34]. The median weight of the male and female cycles sold in the Stockholm region has been about 18.5 kg for a number of years [13], and adding that weight will lead to an approximate 30% increase in the body + cycle weight of a 60 kg person, but only about 20% for a person weighing 85 kg. Thus, cycle weight is an important factor to include in this measurement.

The differences in maximal oxygen uptake between the individual commuter cyclists and the corresponding values for different ages in the population, based on the regression equations, motivated scaling down the DDR slopes of the cycle commuters with the factors 0.719 for the males and 0.763 for the females. The strong significance in each of these differences (p<0.001) and the concordance in the relative size of this cycle commuter to general population difference between the sexes indicates that the existing cycle commuters constitute a subgroup that is not representative of the general population. Interestingly, this difference is of the same order of magnitude as noted by Grimby and Saltin [35] when well-trained middle-aged and elderly endurance athletes were compared with age-matched population samples. The concordance is somewhat surprising since athletes both train at higher intensities and can be expected to constitute a more positive selection within the population. Part of the explanation for this can be the increased body weights within the general population in a short period of time, and thereby a widening gap between exercising and more sedentary samples within the population. For the years between 1990 and 2015, our calculations point to secular body weight increases ranging between 2.8 and 7.0 kg for different age groups of males, and 3.0–6.0 kg for females. If we extrapolate these changes in body weight back to the calender year 0 (Anno Domini), i.e., the value representing the y-intercept, we obtain negative body weights in the range of -45 –-455 kg in different age groups (cf. Table 4). This illustrates the nonsustainability of the secular trends in population body weight in our times. The body weights for the commuter bicyclists were sampled during the period 2006–2007, but the secular weight development described above was not applied to this group since cycle commuting appears to sustain body weights effectively over time [36,37].

It can be inferred that if a sample within the general population became cycle commuters, their physical work capacity would increase and their DDR would approach or mimic that of the existing cycle commuters. Studies following the effect of commencing commuter cycling at modest volumes for periods of up to a year show, however, small changes in various indicators of physical work capacity [38,39,40]. Furthermore, drop-out analyses have pointed out that both population samples studied by means of the exercise physiology test were more physically active during leisure time than the larger population samples that responded to a questionnaire ([23], p. 23; [24], p. 18). Altogether, the present formulas represent reasonable levels within the normal population, and this also refers to samples within the population that had started to cycle commute at modest levels.

The final step in the formation of the formulas relates to the decrease in aerobic power with increasing age, cf. ([17]; [41], p. 319). This is mirrored in decreasing commuter cycling speeds with age in both males and females [13], which is in line with the fact that physical training does not prevent a decrease in the maximal oxygen uptake with age, as has been demonstrated in well-trained middle-aged and elderly endurance athletes [35]. With increasing age, there might be a selective drop-out of commuter cycling individuals with lower maximal oxygen uptakes. Therefore, we based the age correction factor on the general population samples from 1990 and 2000 with updated body weights and cycle weights included. Between the ages of 20 and 65, a 48% decrease in relative oxygen uptake levels among the males and a 43% decrease among the females were noted. This age effect is slightly greater than the approximate 39% decreases in determined maximal oxygen uptake for males and 37% for females noted in the general Swedish population in the 1950s–1960s, as described by Åstrand and Rodahl ([18], p. 311). This might be due to different relative changes in BMI or levels of physical activity and exercise or training in different age groups.

Issues concerning the external validity of the formulas also need to be discussed. Firstly, note that, as stated in Methods, the cycle commuters had bicycles which most commonly had more than five gears, and that the percentage of such cycles for the males was 78% and for the females 56%. Secondly, some comments about the areas cycled in. As stated in Methods, the landscape in Greater Stockholm is generally rather flat, but with frequent smaller slopes, and occasionally more demanding hills of up to about 15-meter elevations. This is representative of large parts of Sweden, but there are clearly regional exceptions with both more and less hilliness, which probably affect the DDR. It would therefore be of great value to study these issues in landscapes with varying topographies.

A characteristic affecting the DDR, is whether one cycles in inner urban vs suburban and rural areas, with the latter representing on the average 0.65 km/h higher speeds [13]. Furthermore, the DDRs described are representative for daylight conditions during both the morning and the evening commute, and with no snow or ice on the ground, cf. [19].

Another issue concerning external validity is related to both aerobic fitness and body weight, as well as to the BMI of other populations. The secular changes over the last few decades in many countries with increased body weight and BMI are well known, e.g. [42]. They affect the DDR [13]. In, for example, the USA, an average increase in body weight of 10 kg has been noted during a 30-year period, and the prevalence of overweight and obesity has risen from about 10% to over 35% [43, 44]. This illustrates the need to adapt the present formulas for DDR to the specific population of interest. For such comparisons, the basis for our study in terms of anthropometric measures is stated in Tables 5 and 6.

The last issue of external validity is related to the possible effect on DDR of the density of cyclists along a cycle path. At velocities over 15 km · h^−1^ on level ground, the wind resistance can become the major resistive force in cycling [45]. Following behind another cyclist can lower the required power output with up to about 40% at high cycling velocities [46]. This could facilitate higher velocities in general. However, a higher density of cyclists may also lead to an adjustment of the average speed to those accomplished by individuals with lower levels of maximal oxygen uptake. The consequence of this is that the DDR may vary with the density of commuter cyclists along a certain route. In Greater Stockholm, only about 7% of the commuting trips used cycling as the main modality in 2004 and during the same season as when the data for establishing the DDR were gathered ([20], p. 25). It would therefore be interesting to study these matters in cities with dense flows of cycle commuters, such as Copenhagen and Amsterdam.

## Conclusions

In conclusion, we present the first estimations of duration-distance relations for cycle commuting for adult males and females in a general population. The bases for these estimations are: (i) data from commuter cycling in Greater Stockholm, Sweden, under day light conditions and with no snow or ice on the ground, and (ii) data on body weight, age and maximal oxygen uptake in cycle commuters and in the general population. Formulas for these relations in the normal Swedish male and female populations of 2015 are presented. They enable reasonable evaluations of the extent to which commuting distances can be cycled within certain durations. Realistic evaluations of cycle commuting from various public health perspectives are thereby facilitated.

## Supporting information

S1 Methods The Physically Active Commuting in Greater Stockholm Questionnaire 1 (PACS Q1). The original version in Swedish.
(PDF)

S2 Methods The Physically Active Commuting in Greater Stockholm Questionnaire 1 (PACS Q1). The original version in Swedish translated into English.
(PDF)

S3 Methods Instructions for how to fill in the Physically Active Commuting in Greater Stockholm Questionnaire 1 (PACS Q1) and maps. The original version in Swedish.
(PDF)

S4 Methods Instructions for how to fill in the Physically Active Commuting in Greater Stockholm Questionnaire 1 (PACS Q1) and maps. The original version in Swedish, translated into English.
(PDF)
