# Correction: Estimating duration-distance relations in cycle commuting in the general population

**DOI:** 10.1371/journal.pone.0218221

**Published:** 2019-06-05

**Authors:** Peter Schantz, Lina Wahlgren, Jane Salier Eriksson, Johan Nilsson Sommar, Hans Rosdahl

There is an error in the ninth, tenth, and eleventh sentence of the Abstract. The correct sentences are: The empirical formula for the distance (*D*, *km*) was based on duration (*T*, *hours*) · speed (km/hours) · a correction factor from cycle commuter to the general population · age adjustment (*A*, *years*). For the males in the general population the formula was: *D* = *T* · 20.76 km/h · 0.719 · (1.676–0.01476 · *A*). For females, the formula was: *D* = *T* · 16.14 km/h · 0.763 · (1.604–0.01288 · *A*).

In the Results, there is an error in the second sentence of the second paragraph. The correct sentence is: For males, the age correction factor was (1.676–0.01476 · *A*), where *A* stands for age (years) and, for the females, it was (1.604–0.01288 ·*A*).

In the Results, there is an error in the second and third sentence of the third paragraph. The correct sentence is: The formula estimating the distance (*D*, *km*) based on duration (*T*, *hours*) and age (*A*, *years*) for males in the population in the age span of 20–65 years was: *D* = *T* · 20.76 km/h · 0.719 · (1.676–0.01476 · *A*), where the factor 0.719 reflects the cycle commuter-to-population effect. For females in the same age span, the formula was: *D* = *T* · 16.14 km/h · 0.763 · (1.604–0.01288 · *A*), where 0.763 reflects the cycle commuter to general population effect.
