# Correction: Correction: Estimating duration-distance relations in cycle commuting in the general population

**DOI:** 10.1371/journal.pone.0218866

**Published:** 2019-06-19

**Authors:** Peter Schantz, Lina Wahlgren, Jane Salier Eriksson, Johan Nilsson Sommar, Hans Rosdahl

There is an error in the first sentence of the second paragraph. The correct sentence is: In the Results, under the heading “The estimated duration-distance relation for the general Swedish population in 2015,” there is an error in the second sentence of the second paragraph.

There are errors in the third paragraph. The correct paragraph is: In the Results, under the same heading as above, there is an error in the second and third sentence of the third paragraph. The correct sentences are: “The formula estimating the distance (*D*, *km*) based on duration (*T*, *hours*) and age (*A*, *years*) for males in the population in the age span of 20–65 years was: *D* = *T*·20.76 km/h·0.719·(1.676−0.01476·*A*), where the factor 0.719 reflects the cycle commuter-to-population effect. For females in the same age span, the formula was: *D* = *T*·16.14 km/h·0.763·(1.604−0.01288·*A*), where 0.763 reflects the cycle commuter to general population effect.”
