# Supplementary material for: Estimating duration-distance relations in cycle commuting in the general population
Source: PLoS One. 2018 Nov 16;13(11):e0207573. doi: 10.1371/journal.pone.0207573 (PMC6239310; doi:10.1371/journal.pone.0207573)
Supplement: S2 Methods — The original version in Swedish translated into English. (PDF) [file pone.0207573.s002.pdf]

Translation from Swedish, 2018-06-27.

Survey for those who walk or bike all the way to their place of work/study

General questions

1. **Your gender – female or male?** ☐ Female ☐ Male

2. **In what year were you born**? 19 ☐☐

3. **What is your weight?** Please answer in full kg. ☐☐☐ kg

4. **How tall are you?** ☐☐☐ cm

5. **Are you**:
   - **gainfully employed?** ☐
   - **a volunteer?** ☐
   - **a student?** ☐
   - **Other?** ☐

   please specify:…………………..…..

6. **Do you have access to a shower at your place of work/study?**

   ☐ Yes, conveniently ☐ Yes, but not conveniently ☐ No ☐ Don’t know

If you walk all the way to your place of work/study but never use a bike to get there, proceed to question no. 18 on page 5.

Translation from Swedish, 2018-06-27.

Questions concerning your bike ride to your place of work/study

7. **What is your usual route to and from your place of work/study?** Indicate on the map provided. Follow the attached letter of instruction.

8. **Estimate the length of your route.** Indicate the approximate distance in kilometres. You may use one decimal (e.g. 600 meters = 0.6 km).

   ☐☐. ☐ km

9. **How much time does the bike ride usually take <u>from your home</u> to your place of work/study?** Measure the time of the ride on an ordinary day when you do not do errands along the way.

   ☐ Hours ☐☐ Minutes

10. **How much time does the bike ride usually take <u>from your place of work/study</u> to your home?** Measure the time for the ride on an ordinary day when you do not do errands along the way.

    ☐ Hours ☐☐ Minutes

11. **On average, how strenuous do you usually experience your bike ride to your place of work/study?** Mark with <u>one</u> x in <u>each</u> column at a digit on the 6 – 20 scale.

<u>From your home</u> to your place of work/study

☐ 6
☐ 7 Very, very light
☐ 8
☐ 9 Very light
☐ 10
☐ 11 Fairly light
☐ 12
☐ 13 Somewhat hard
☐ 14
☐ 15 Hard
☐ 16
☐ 17 Very hard
☐ 18
☐ 19 Very, very hard
☐ 20

<u>From place of work/study</u> to your home

☐ 6
☐ 7 Very, very light
☐ 8
☐ 9 Very light
☐ 10
☐ 11 Fairly light
☐ 12
☐ 13 Somewhat hard
☐ 14
☐ 15 Hard
☐ 16
☐ 17 Very hard
☐ 18
☐ 19 Very, very hard
☐ 20

12. **Do you usually bike to and from your place of work/study on the same day?** I.e. you do not leave your bike at your place of work/study.

☐ Yes ☐ No ☐ Don't know

If you answered No, explain why: ……………………………………

…………………………………………………………………………..………………..

The following is a question, which, combined with the routes you draw on the map, will provide a valuable picture of your physical activity as well as the potential health effects of your biking.

13. **How many bike rides (see instruction below) do you make between your home and your place of work/study on average per week during different months?** Mark once (x) for each month.

This is how you fill in your answers:

- **If you bike to and from your place of work/study 5 days a week during the whole month, the number of bike rides per weeks will be 10 per week on average.**
- **If, instead, you have half the month off, the number of bike rides will be on average 5 per week during that month.**
- **If you are on holiday the whole month, the number of bike rides will be 0.**
- **If, on average, you make fewer than 1 bike ride per week but in all more than 0, you shall mark the <1 box.**
- **If you bike to and/or from your place of work/study irregularly and on few occasions during the year, and you are uncertain about which months you do so, mark the "*Different alternative*" box.**

**Month**	**Average number of return bike rides per week**																		
	0	<1	1	2	3	4	5	6	7	8	9	10	11	12	13	14	More...	State number:	Don't know
**January**	☐	☐	☐	☐	☐	☐	☐	☐	☐	☐	☐	☐	☐	☐	☐	☐	☐	…..	☐
**February**	☐	☐	☐	☐	☐	☐	☐	☐	☐	☐	☐	☐	☐	☐	☐	☐	☐	…..	☐
**March**	☐	☐	☐	☐	☐	☐	☐	☐	☐	☐	☐	☐	☐	☐	☐	☐	☐	…..	☐
**April**	☐	☐	☐	☐	☐	☐	☐	☐	☐	☐	☐	☐	☐	☐	☐	☐	☐	…..	☐
**May**	☐	☐	☐	☐	☐	☐	☐	☐	☐	☐	☐	☐	☐	☐	☐	☐	☐	…..	☐
**June**	☐	☐	☐	☐	☐	☐	☐	☐	☐	☐	☐	☐	☐	☐	☐	☐	☐	…..	☐
**July**	☐	☐	☐	☐	☐	☐	☐	☐	☐	☐	☐	☐	☐	☐	☐	☐	☐	…..	☐
**August**	☐	☐	☐	☐	☐	☐	☐	☐	☐	☐	☐	☐	☐	☐	☐	☐	☐	…..	☐
**September**	☐	☐	☐	☐	☐	☐	☐	☐	☐	☐	☐	☐	☐	☐	☐	☐	☐	…..	☐
**October**	☐	☐	☐	☐	☐	☐	☐	☐	☐	☐	☐	☐	☐	☐	☐	☐	☐	…..	☐
**November**	☐	☐	☐	☐	☐	☐	☐	☐	☐	☐	☐	☐	☐	☐	☐	☐	☐	…..	☐
**December**	☐	☐	☐	☐	☐	☐	☐	☐	☐	☐	☐	☐	☐	☐	☐	☐	☐	…..	☐

☐ **Different alternative**

14. **How many times do you usually have to stop at red lights during your ride to your place of work/study?** You may count the number of stops you make during a normal day.

☐ I do not stop
☐ I stop. State the number of stops:

☐ 1	☐ 2	☐ 3	☐ 4	☐ 5	☐ 6
☐ 7	☐ 8	☐ 9	☐ 10	☐ 11	☐ 12
☐ 13	☐ 14	☐ 15	☐ 16	☐ 17	☐ 18
☐ 19	☐ 20	☐ More than 20 times, state number……			
☐ Don't know					

15. **Do you become sweaty when you bike <u>to</u> your place of work/study?**

☐ No, never
☐ Yes, 1-25 % of the times
☐ Yes, 26-50 % of the times
☐ Yes, 51-75 % of the times
☐ Yes, 76-100 % of the times
☐ Don't know

16. **Do you usually shower after biking to your place of work/study?**

☐ No, never
☐ Yes, 1-25 % of the times
☐ Yes, 26-50 % of the times
☐ Yes, 51-75 % of the times
☐ Yes, 76-100 % of the times
☐ Don't know

Question about your bicycle

17. **What type of bicycle do you use to bike to your place of work/study?**

☐ Bicycle without gears
☐ Bicycle with gears (2- 4 gears)
☐ Bicycle with gears (5 gears or more)
☐ Don't know

Proceed to the next question if, during the last year, you have on some occasion walked all the way to your place of work/study. If not, proceed to question number 28 on page 8.

Questions about your walk to your place of work/study

18. **What is your most frequent route to and from your place of work/study?** Indicate this on the map provided. Follow the attached letter of instruction.

19. **Estimate how long your route is.** State the approximate distance in kilometres. You may use one decimal (e.g. 600 meters = 0.6 km)

☐☐. ☐ km

20. **How much time does the walk usually take <u>from your home</u> to your place of work/study?** Measure the time for the route on an ordinary day when you do not do any errands along the way.

☐ Hours ☐☐ Minutes

21. **How much time does the walk usually take <u>from your place of work/study</u> to your home?** Measure the time for the route on an ordinary day when you do not do any errands along the way.

☐ Hours ☐☐ Minutes

22. **On average, how strenuous do you experience your walk to your place of work/study?** Place <u>one</u> x in <u>each</u> column against a digit on the 6 – 20 scale.

<u>From your home to your place of work/study</u>

☐ 6
☐ 7 Very, very light
☐ 8
☐ 9 Very light
☐ 10
☐ 11 Fairly light
☐ 12
☐ 13 Somewhat hard
☐ 14
☐ 15 Hard
☐ 16
☐ 17 Very hard
☐ 18
☐ 19 Very, very hard
☐ 20

<u>From your place of work/study to your home</u>

☐ 6
☐ 7 Very, very light
☐ 8
☐ 9 Very light
☐ 10
☐ 11 Fairly light
☐ 12
☐ 13 Somewhat hard
☐ 14
☐ 15 Hard
☐ 16
☐ 17 Very hard
☐ 18
☐ 19 Very, very hard
☐ 20

23. **Do you usually walk to and from your place of work/study during the same day?**

☐ Yes ☐ No ☐ Don' know

If you have answered No, explain why: ……………………………………………
……………………………………………………………………………………………

> The following is a question, which, combined with the routes you draw on the map, will provide a valuable picture of your physical activity as well as potential health effects of your walking.

24. **How many times do you walk (see instruction below) between your home and your place of work/study, on average, per week during each month?** Tick once (x) for each month.

> ***This is how you fill in your answers:***
> - **If you walk to and from your place of work/study 5 days a week during the whole month the number of walks will be on average 10 per week.**
> - **If, instead, you have half the month off, the number of walks will be, on average, 5 per week during that month.**
> - **If you are on holiday the whole month, the number of walks will be 0.**
> - **If, on average, you walk fewer than 1 time per week but in total more than 0, mark the <1 box.**
> - **If you walk to and/or back from your place of work/study irregularly and on a few occasions during the year, and you are uncertain about which months you do so, mark the "*Different alternative*" box".**

**Month**	**Number of walks on average per week**																		
	0	<1	1	2	3	4	5	6	7	8	9	10	11	12	13	14	More…	State number:	Don't know
**January**	☐	☐	☐	☐	☐	☐	☐	☐	☐	☐	☐	☐	☐	☐	☐	☐	☐	…...	☐
**February**	☐	☐	☐	☐	☐	☐	☐	☐	☐	☐	☐	☐	☐	☐	☐	☐	☐	…...	☐
**March**	☐	☐	☐	☐	☐	☐	☐	☐	☐	☐	☐	☐	☐	☐	☐	☐	☐	…...	☐
**April**	☐	☐	☐	☐	☐	☐	☐	☐	☐	☐	☐	☐	☐	☐	☐	☐	☐	…...	☐
**May**	☐	☐	☐	☐	☐	☐	☐	☐	☐	☐	☐	☐	☐	☐	☐	☐	☐	…...	☐
**June**	☐	☐	☐	☐	☐	☐	☐	☐	☐	☐	☐	☐	☐	☐	☐	☐	☐	…...	☐
**July**	☐	☐	☐	☐	☐	☐	☐	☐	☐	☐	☐	☐	☐	☐	☐	☐	☐	…...	☐
**August**	☐	☐	☐	☐	☐	☐	☐	☐	☐	☐	☐	☐	☐	☐	☐	☐	☐	…...	☐
**September**	☐	☐	☐	☐	☐	☐	☐	☐	☐	☐	☐	☐	☐	☐	☐	☐	☐	…...	☐
**October**	☐	☐	☐	☐	☐	☐	☐	☐	☐	☐	☐	☐	☐	☐	☐	☐	☐	…...	☐
**November**	☐	☐	☐	☐	☐	☐	☐	☐	☐	☐	☐	☐	☐	☐	☐	☐	☐	…...	☐
**December**	☐	☐	☐	☐	☐	☐	☐	☐	☐	☐	☐	☐	☐	☐	☐	☐	☐	…...	☐

☐ Different alternative

25. **How many times do you usually have to stop at red lights during your walk to your place of work/study?** You may count the number of stops you make during an ordinary day.

☐ I do not stop
☐ I stop. State the number of stops:

☐ 1	☐ 2	☐ 3	☐ 4	☐ 5	☐ 6
☐ 7	☐ 8	☐ 9	☐ 10	☐ 11	☐ 12
☐ 13	☐ 14	☐ 15	☐ 16	☐ 17	☐ 18
☐ 19	☐ 20	☐ More than 20 times. State the number…….			
☐ Don’t know					

26. **Do you become sweaty when you walk to your place of work/study?**

☐ No, never
☐ Yes, 1-25 % of the times
☐ Yes, 26-50 % of the times
☐ Yes, 51-75 % of the times
☐ Yes, 76-100 % of the times
☐ Don’t know

27. **Do you usually shower after walking <u>to</u> your place of work/study?**

☐ No, never
☐ Yes, 1-25 of the times
☐ Yes, 26-50 % of the times
☐ Yes, 51-75 % of the times
☐ Yes, 76-100 % of the times
☐ Don’t know

Questions about your physical activity during working/studying hours

28. **How physically strenuous has your daily work or your daily occupation (not spare time) been during the last 12 months?**

☐ Very easy, predominantly sedentary

☐ Light physical work, but I do move a good deal (for example light industrial work, shop assistant, teacher)

☐ Physically rather strenuous work (for example cleaner, postman, assistant nurse)

☐ Physically very strenuous work (heavy manual work, for example bicycle messenger, heavy forestry work, or construction work)

29. **Is it possible for you to do physical exercise during paid working hours?**

☐ No
☐ Yes, but I do not take the opportunity to do so
☐ Yes, and I make use of the opportunity
☐ Don't know

If you have answered "**Yes, and I make use of the opportunity**", proceed with the next question; if not, proceed to question 32 on page 10.

30. **What type of activity, how often and for how long do you usually do physical exercise/training during paid working hours?** Use an average value if it varies between weeks. You may indicate several activities.

**Activity**	**Number of occasions per week**	**Time per training session**
☐ Weight training	…………… times	…….. min
☐ Fitness training	…………… times	…….. min
☐ Ball game	…………… times	…….. min
☐ Keep-fit exercise (e.g. aerobics/Friskis & Svettis)	…………… times	…….. min
☐ Other, indicate what:		
………………………..…	…………… times	…….. min
………………………..…	…………… times	…….. min
………………………..…	…………… times	…….. min

31. **On what average level of intensity do you usually exercise/train during paid working hours?** Mark (x) only once. Thus, use an average value for the different activities if you practise different kinds of activities.

- ☐ 6
- ☐ 7 Very, very light
- ☐ 8
- ☐ 9 Very light
- ☐ 10
- ☐ 11 Fairly light
- ☐ 12
- ☐ 13 Somewhat hard
- ☐ 14
- ☐ 15 Hard
- ☐ 16
- ☐ 17 Very hard
- ☐ 18
- ☐ 19 Very, very hard
- ☐ 20

Questions about your physical activity during your spare time, except commuting time

32. **How much have you generally moved or exerted yourself physically in your *spare time* during the last year? NB! Mark (x) *<u>all</u>* options that apply to you.** Do not include physical activity during commuting, i.e. biking and walking to your place of work/study.

☐ a) I have moved very little.

☐ b) I have moved very little but sometimes taken an odd walk or something like that.

☐ c) I have had "everyday exercise" in connection with cleaning, climbing stairs, garden work, social dancing, strolling or light bike rides (except walking/biking all the way to the place of work/study), taking the dog for a walk etc.

☐ d) I have, in addition to the activities in c), devoted myself to some light kind of exercise, like strolls (or other activities with similar exertion) *at least once every week*.

☐ e) I have devoted myself to more strenuous exercise, such as quick walks, jogging, swimming, keep-fit exercise or equivalent *at least once every week*.

☐ f) I have regularly devoted myself to *hard training or competition* where the physical exertion has been great, e.g. running and various ball games.

If you have marked the alternatives e) and/or f) proceed with the following question. Others may proceed to question number 35 on the next page.

33. **If you marked the alternatives e) and/or f) in question number 32, what type of activities, how often and for how long do you usually exercise/train?** Use an average value if it varies from one week to another. You may indicate several activities.

**Activity**	**Category e)**	**f)**	**Time per occasion**	**Number of times per week**	**No of months per year**
☐ Weight training	☐	☐	……… min	………....	…………
☐ Fitness training	☐	☐	……… min	………....	…………
☐ Ball games	☐	☐	……… min	………....	…………
☐ Keep-fit exercise (e.g. aerobics/ Friskis & Svettis)	☐	☐	……… min	………....	…………
☐ Other, indicate what:					
………………	☐	☐	……… min	………....	…………
………………	☐	☐	……… min	………....	…………

34. **If you marked the alternatives e) and/or f) of question number 32 and 33, on what average level of exertion do you usually exercise/train?**
Mark only one option in each column. Thus, use an average value for the various activities if you do several activities within the alternatives e) and f), respectively.

e) f)
☐ ☐ 6
☐ ☐ 7 Very, very light
☐ ☐ 8
☐ ☐ 9 Very light
☐ ☐ 10
☐ ☐ 11 Fairly light
☐ ☐ 12
☐ ☐ 13 Somewhat hard
☐ ☐ 14
☐ ☐ 15 Hard
☐ ☐ 16
☐ ☐ 17 Very hard
☐ ☐ 18
☐ ☐ 19 Very, very hard
☐ ☐ 20

35. **Are you interested in participating in the next stage of this study?**

Stage 2. Second part of the survey ☐ Yes ☐ No ☐ Don’t know

Stage 3. Fitness test and measuring energy metabolism during the route. ☐ Yes ☐ No ☐ Don’t know

36. **If you have any comments on this inquiry and its question, you are welcome to write them here and, if necessary, to continue on the back page.**

____
____
____
____
____
____
____
____
____
____

Many thanks for your help!
