# Supplementary material for: Estimating duration-distance relations in cycle commuting in the general population
Source: PLoS One. 2018 Nov 16;13(11):e0207573. doi: 10.1371/journal.pone.0207573 (PMC6239310; doi:10.1371/journal.pone.0207573)
Supplement: S4 Methods — The original version in Swedish, translated into English. (PDF) [file pone.0207573.s004.pdf]

Translation from Swedish 2018-06-28

Instructions for filling in the questionnaire and the map

Indicate your answer in the box with an x, like this: ☒. If you mark the wrong box, then fill the whole box with colour, like this: ■ Then tick the correct box.

The questions about your commuting refer to your travels during the last year to your ordinary place of work/study. When you answer the questions, consider the days when you do not do any errands along the way.

To fill in the questionnaire, you need to do four simple things:

1. Measure the time it takes to bike/walk both to and from your place of work/study (see questions 9,10, 20, 21),
2. Count the number of stops at red lights that you usually make along the route to your place of work/study (see questions 14 and 25),
3. Estimate your average level of exertion during the trip (see alternative answers to question 11 and 22), and
4. Indicate your route between your place of residence and your place of work/study on the attached map, in accordance with the instruction below.

Information about your travel route is of great importance for the study. If you feel uncertain about the route you bike/walk, we recommend that you bike/walk the route and register the names of the streets.

1. Mark your usual route to your place of work/study with an unbroken line for biking ___________ and a line with crosses for walking –X—X—X–. Indicate your residence with a **B** and your place of work with ■ (see figures 1 and 2). Preferably use a blue or red fountain pen or ball pen. Thus, you shall mark both biking and walking, if at times you bike and at times you walk.

2. If your route back home from your place of work/study deviates from the route you take there, we ask you to draw a line with circles for your way home if you bike –O—O—O– and a line with triangles if you walk ↗—↗—↗ (see figures 1 and 2).

3. If you usually take your children to preschool/school we ask you to indicate the location of the preschool/school with an **S** on the map (see figures 1 and 2). Exclude the time it takes to deliver the children from the travelling time.

4. If you walk or bike along park alleys, in tunnels or along paths which are not shown on the map, we ask you to draw the route on the map as carefully as possible.

5. If you have two places of work/study, we ask you to indicate the route to and from the place of work/study where you spend most of the time. If you work/study just as much in two or more places we ask you to indicate only the route to <u>one</u> of the places of work/study.

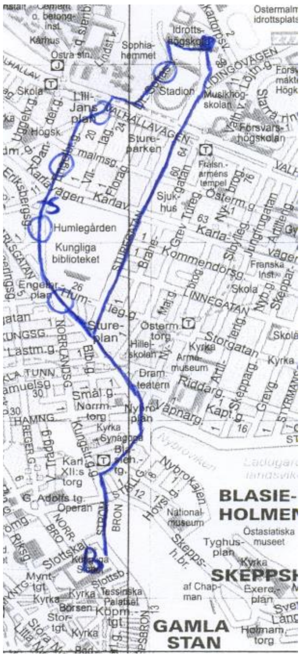

Figure 1. Example of how to indicate a biking route on the map.

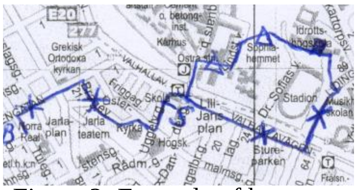
Figure 2. Example of how to indicate a walking route on the map.

The attached map has usually been taken from the phone book. If it does not make sense, you can either amend it, using the Yellow Pages map, or contact us (see below), and we will send you a new map. If you have questions, you are welcome to call 08-16 14 53 during daytime.

FoU-gruppen Rörelse, hälsa och miljö, Åstrandlaboratoriet, Idrottshögskolan i Stockholm, Box 5626, 114 86 Stockholm
epost: erik.stigell@ihs.se tel: 08-16 14 53 fax: 08-660 75 11
